# Breaking the plastic habit: Drivers of single-use plastic reduction among Thai university students

**DOI:** 10.1371/journal.pone.0299877

**Published:** 2024-05-09

**Authors:** Oluseye O. Oludoye, Nuta Supakata

**Affiliations:** 1 School of Health and Life Sciences, Teesside University, Middlesbrough, United Kingdom; 2 Department of Environmental Science, Faculty of Science, Chulalongkorn University, Bangkok, Thailand; 3 Research Unit (RU) of Waste Utilization and Ecological Risk Assessment, Chulalongkorn University, Bangkok, Thailand; Guangxi Normal University, CHINA

## Abstract

This study investigated the decision-making dynamics for pro-environmental behavior among Thai university students, focusing on reducing the consumption of single-use plastics (SUP). By adopting a dynamic approach to the Theory of Planned Behavior (TPB), the research examined the influence of psychosocial factors, including attitudes, perceived behavioral control, and subjective norms, on SUP reduction intention at different phases of behavior change. Using structural equation modelling, we analyzed quantitative data (n = 317) from the selected universities. The results revealed that attitudes predicted behavioral intentions only among individuals in the contemplation phase of reducing SUP. Attitudes had a small but limited influence on the behavioral intentions of students who had not yet acted. Perceived behavioral control, on the other hand, significantly impacted behavioral intentions across all phases of behavior change, highlighting its importance in SUP reduction. The study also confirmed subjective norms’ positive influence on students’ behavioral intentions in the pre-contemplation phase. Practical implications suggested segmenting residents based on their behavior change phase so that public policymakers can allocate resources more efficiently and effectively by tailoring campaigns to specific behavior change phases, ultimately promoting sustainable behavior among university students.

## 1.0 Introduction

Single-use plastic (SUP) packaging has become increasingly prevalent worldwide, providing convenience for people in various aspects of their lives. This ease of use, however, has had a major impact on the natural world. Global plastic production skyrocketed from 2 million tons in 1950 to 381 million tons in 2015—packaging accounts for the largest share, comprising approximately 36% of total SUP production [1]. The proliferation of SUP packaging directly threatens the environment, particularly our oceans, as plastic materials are notoriously difficult to degrade. Numerous efforts have been made to address plastic waste problems in response to this pressing issue. These include initiatives such as waste reduction, sorting and plastic recycling [2–4], implementing bans and levies on plastic carrier bags [4, 5], and exploring the use of alternatives such as biodegradable plastics [6]. While these measures are crucial steps towards mitigating the plastic waste crisis, their effectiveness relies heavily on the awareness and understanding of the public. Therefore, raising public awareness regarding the detrimental impact of plastic-based problems is imperative. Without widespread awareness and knowledge, even the most well-intentioned efforts would fall short of their desired outcomes. Educating individuals about the environmental consequences of plastic usage can foster a sense of responsibility and encourage sustainable practices that minimize SUP waste.

Educational institutions are crucial in nurturing a generation that values and safeguards the environment. Through tailored educational approaches, these institutions play a pivotal role in promoting environmental knowledge, behavior, and engagement among students at various educational levels and across different countries. Many sustainable solutions worldwide have been linked to education [7, 8]. Young people are frequently at the forefront of awareness campaigns as critical stakeholders who hold the key to future sustainability goals [9]. University communities are responsible for ensuring long-term practices by educating people who could hold key positions in society [10]. As a result, universities are responsible for ensuring that young people understand the importance of sustainable practices such as plastic waste management. Universities provide these students with the knowledge, tools, and technology needed to promote pro-environmental behaviors in their communities [11]. This study focuses on young elite Thais at selected institutions because they are essential stakeholders in Thailand’s waste management reduction efforts [12]. Thai youths are active social agents with the potential to significantly contribute to society [13].

Thailand faces difficulty reducing municipal solid waste (MSW) from urban consumption due to domestic waste management regulations that have been criticized for failing to adapt to long-term public participation and encouraging pro-environmental behaviors [14, 15]. National MSW management schemes now incorporate the 3Rs, and the waste management hierarchy recommends putting an emphasis on waste prevention or reduction first, then source reuse, and finally, recycling and disposal [16]. Campaign methods, on the other hand, are more likely to center on the recycling phase [15]. Even though plastic cups, food containers, and straws are reported to be a major problem at waste management facilities and in marine debris, there is a lack of policy interventions targeting the reduction of these SUP products in consumption-related activities [17].

Waste management relies heavily on the actions and understanding of today’s college students. As shown by the research, students’ environmental behavior [18, 19] is the primary factor in the success of waste management, followed by an increase in their knowledge [20]. Evidence from studies by Goldman, Ayalon [21] and others shows that students’ active participation in environmental issues can help bring about a zero waste society (i.e., with no trash to throw away). Important determinants of waste management include people’s attitudes, social norms, and ability to exercise self-control [22]. Environmental education in the context of higher education imparts information, shapes students’ perspectives on waste management, and guides them toward more effective plans for and execution of waste management [23].

Many existing studies have focused on plastic waste management [5, 24–29] but not among university students, especially within the framework of the theory of planned behavior (TPB) and the transtheoretical model (TTM) of change. Such studies are critical because students are intellectual forerunners and thus serve as a model for others [30]. They represent a community that significantly influences a larger society, and their responses to waste management problems before entering professional careers would reflect the values and beliefs instilled in them since childhood. Not everyone is at the same level of SUP reduction readiness. As a result, it is worthwhile to investigate the various stages of readiness and the vital psychosocial factors underlying each stage.

Understanding the factors that draw young people into waste management is a crucial scientific endeavor and practical concern for developing a sustainable system. Given those mentioned above, this study aimed to identify attitudes, beliefs and motivational processes that might be useful in designing interventions to reduce SUP among university students in Thailand using the integrated model of the TPB and TTM.

### 1.1 Theoretical underpinnings and hypotheses development

This study is grounded in two key theoretical frameworks: the Theory of Planned Behavior (TPB) and the Transtheoretical Model of Change (TTM).

#### 1.1.1 Psychosocial determinants of pro-environmental behaviours: The TPB

The TPB is a well-established framework used to explain intentions and behavior changes related to environmental and pro-environmental actions [31–35]. It focuses on three primary psychosocial determinants: attitudes, subjective norms, and perceived behavioral control. These factors collectively influence an individual’s intention to engage in a specific behavior, with a positive attitude leading to stronger behavioral intentions. The TPB has found empirical support in various pro-environmental contexts, including waste disposal [35], technology adoption [31], sustainable mining [34], and waste sorting [32].

#### 1.1.2 Transtheoretical Model of Change (TTM)

The TTM, based on Kurt Lewin’s theory, offers a dynamic perspective on behavior change. It posits that successful behavior change involves both motivation (primary stages) and the acquisition of skills and strategies (secondary stages) [36]. While initially developed for health behavior change [37, 38], recent research has explored its applicability to pro-environmental behavior [36, 39, 40]. This model acknowledges the dynamic nature of human behavior and the procedural character of behavior change. For this study, we adopt Bamberg’s and Watakakosol’s transtheoretical models [36, 39–43], which provide a framework for understanding the stages of change, from pre-contemplation to maintenance (Table 1).

**Table 1:** Psychosocial factors and their phases [36, 39, 40].

	Phase	Factors influencing stages of change/phase transition	Cognitive challenge for progressing to the next stage
1	Pre-contemplation	Subjective Norms, Perceived Behavioral Control	Behavioral re-assessment
2	Contemplation	Attitude, Perceived Behavioral Control	Choosing a novel behavioral option
3	Action	Perceived Behavioral Control	Execution of the novel behavior
4	Maintenance	--	Establishment of the newly adopted behavior as a habit

#### 1.1.3 Integration of TTM and TPB

While the TPB provides essential insights into what influences behavioral intentions, it does not explain how behaviors change, such as SUP reduction. The components of TPB, namely attitude, subjective norms, perceived behavioral control, and intention, could theoretically be integrated into the TTM to explain the influencing factors that differentiate one stage from the other [44]. Although TTM-TPB integration is common in health and well-being domain, its use in pro-environmental behavior studies is rare. The number of studies, however, is limited, particularly in the Asian context [39, 44, 45] and none regarding plastic waste management. As a result, the behavioral intentions of youths to reduce single-use packaging need to be investigated by integrating TPB and TTM. The proposed integrated model could investigate students’ SUP reduction based on their readiness and adoption stages and identify the underlying drivers at each stage. Based on the TTM, the conceptual framework emphasizes consumer heterogeneity by proposing that different consumers are at different stages of SUP reduction readiness and adoption [46]. The framework also assumes that incorporating the TPB constructs would make the underlying drivers more salient at different stages.

Despite TPB’s effectiveness and broad application, critics have claimed that its nature is essentially static [40, 42], in that it does not account for the heterogeneity and subtlety of an individual’s preparedness and adoption of a behavior. TTM is regarded as more organic and flexible. The model implies that different individuals are at various behavioral adoption phases and may progress or regress to earlier stages [39, 47]. The TTM was originally a comprehensive model that explained not just the transitory nature of the stages but also the detailed mechanisms underpinning each stage of behavioral change. However, the parsimonious form of the theory has been criticized for failing to explain the underlying variables that drive the phases of change [45]. TTM has been applied to model consumers’ readiness to change regarding SUP reduction. However, no empirical study has integrated TPB and TTM regarding SUP reduction in the literature. Therefore, this study integrated the TPB and TTM to examine the predictors for adopting SUP reduction among Thai students’ population.

**Fig 1:**
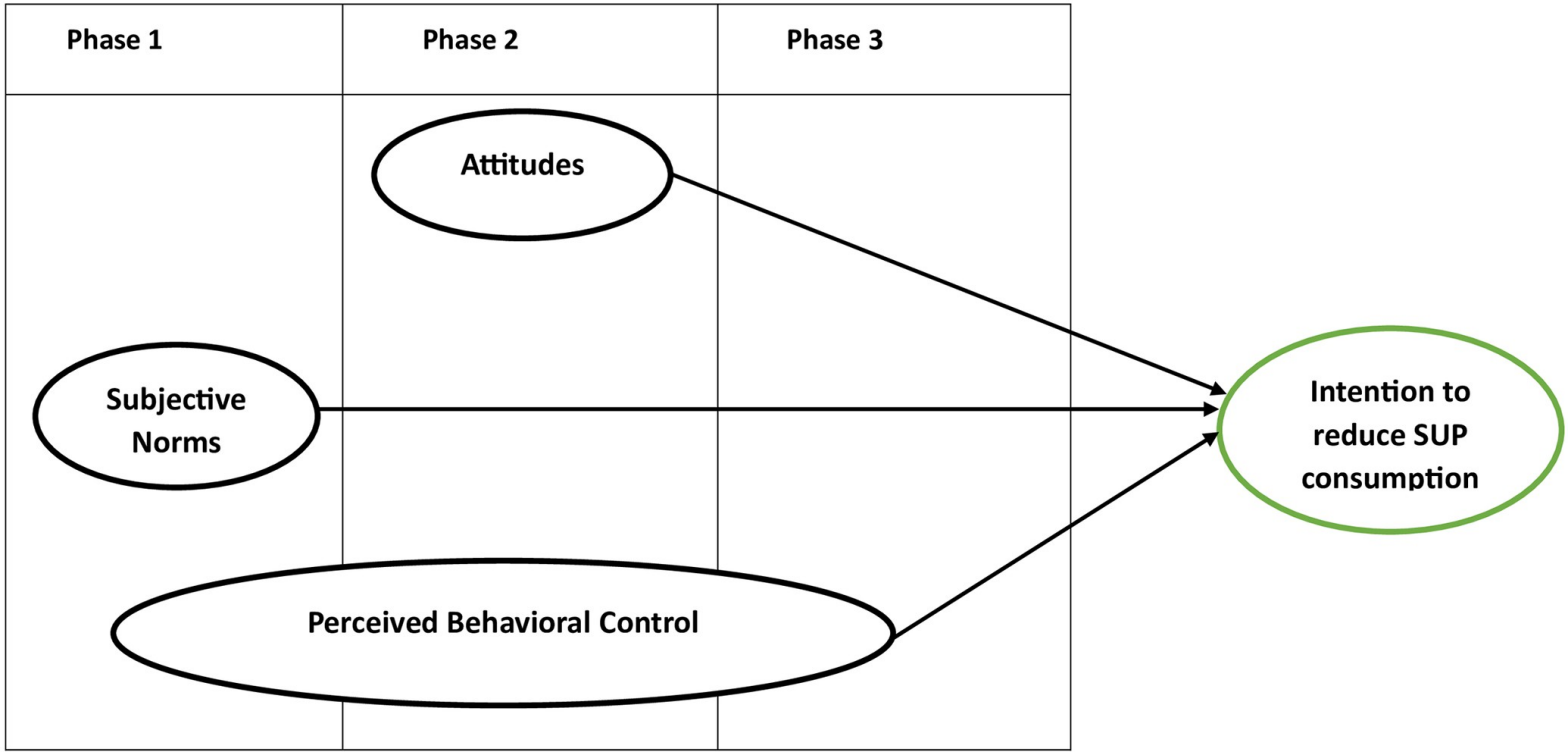
Psychosocial factors within a dynamic phase of behaviour change model.

In the pre-contemplation phase (Phase 1) of behavior change, the primary psychological task is to re-evaluate one’s current and habitual behavior. The objective is to motivate individuals to consider their behavior’s personal and collective disadvantages. Within this phase, a term “subjective norm” is proposed to influence behavioral intentions. Similarly, individuals will assess the difficulty of engaging in the targeted behavior (perceived behavioral control). Individuals choose a new behavioral strategy during the contemplation phase (Phase 2) after considering the outcomes (attitudes) and viability (perceived behavioral control) of the behavior. The third phase (Phase 3), “action,” focuses on the mental preparation necessary for the desired behavior. This requires thinking about the difficulties and helpful context of the desired behavior. When the third phase is accomplished, the fourth phase can begin. The fourth stage, “maintenance,” focuses on keeping progress from stagnating and reverting to old habits and methods. People work to make the desired new behavior automatic by making it a habit. There are no psychosocial factors directly related to behavioral intentions for people in the maintenance phase because they have completed the entire behavior change process. The proposed relationships are supported by empirical evidence (Table 1), and Bamberg has tested the model in the context of sustainable transportation. Considering Bamberg [36] identifies three distinct phases, Fig 1 provides a dynamic model depicting these interrelationships.

Therefore, the study models/theories (TPB/TTM) explained above lead us to hypothesize that social and psychological factors like attitude, subjective norm, and perceived behavioral control have varying impacts on individuals’ behavioral intentions to cut back on SUP consumption and that these intentions vary across stages. This research zeroed in on stages one through three because those are where plastic use habits are established, and only minor problems with maintaining those habits arise. Therefore, using data from Table 1, we make the following predictions:

**H1a:** In the pre-contemplation phase, the perceived behavioral control positively affects the intention to reduce SUP consumption.
**H1b:** In the pre-contemplation phase, the subjective norm positively influences the intention to decrease SUP consumption.
**H2a:** During the contemplation phase, individuals’ attitudes positively impact their intention to reduce SUP consumption.
**H2b:** In the contemplation phase, individuals’ perceived behavioral control positively influences their intention to reduce SUP consumption.
**H3:** In the action phase, the perceived behavioral control positively influences the behavioral intention to reduce SUP consumption.

Additionally, we propose that the relationship between behavioral intention and perceived behavioral control varies depending on the phase of behavior change. As mentioned earlier, perceived behavioral control is expected to impact all phases.

**H4:** With the increase in the phase of students’ behavioral change, the influence of perceived behavioral control on their intention to reduce SUP consumption increases.

## 2.0 Methods

### 2.1 Sampling and data collection

We collected online data from February 15 to May 14, 2023; we accessed the data for research purposes (e.g. data analysis) on May 16, 2023. The study involved undergraduate students from Chulalongkorn, King Mongkut’s University of Technology North Bangkok, Kasetsart, Mahidol, and Thammasat Universities’ Departments of Environmental Science. Students with a background in environmental science were chosen for the survey based on the findings of a pilot study that helped refine the research protocol. The researchers noted that students with environmental science backgrounds were more knowledgeable about the adverse effects of single-use plastics (SUPs) than those without such backgrounds. On the other hand, most students without environmental science backgrounds reported having limited knowledge of the impact of SUPs on the ecosystem, and some did not finish the questionnaire. Thus, we interviewed students with environmental science backgrounds to improve the response rate. We collected data using Google Forms to ensure maximum participation and sufficient time for interested students to participate. To reach a more significant number of participants in the target population, we distributed the questionnaire through various messaging and communication platforms such as Facebook, Line App, and Email. This method encouraged students’ participation because it did not require face-to-face interaction with the interviewers, which is crucial given Thailand’s current COVID-19 mitigation protocols. Finally, we used purposive sampling to select 317 participants who met the study’s inclusion and exclusion criteria:

Inclusion criteria: Those who

i were 18 years old or older during data collection.
ii could communicate in the Thai language.
iii were studying at the above-named universities in Thailand.
iv were using SUPs for food packaging.

Exclusion criteria: Those who

i had an internet connectivity problem.
ii were not undergraduate students.
iii were not from the environmental science department.
iv were not available for data collection.

To ensure ethical standards and obtain permission to access students’ contact information, we contacted the Department of Environmental Science at the chosen universities for study approval before the survey began. We communicated the objectives and scope of the study to them. After approval, we emailed prospective participants a questionnaire link outlining the study’s purpose, data collection methods, and potential benefits. This email also included information about the participants’ consent. Interested participants approved the informed consent form by clicking the link and completing a four-section questionnaire. The data collection process took approximately three months. To ensure data completeness, we checked the questionnaire for any missing information upon completion of the interview.

### 2.2 Research instrument/measures

This study questionnaire comprised four main sections: In the first section, participants were required to sign an informed consent form before proceeding to the second section, which focused on demographic questions. The third section assessed participants’ SUP reduction behaviors and included questions designed to evaluate the TPB and TTM constructs. The questionnaire scales used in this study were adapted from those utilized by the TPB [48, 49] and the TTM [47] authors.

#### TPB measures

The study’s TPB measures consisted of four scales, including the attitude toward reducing SUPs, subjective norms (perceptions of others’ attitudes towards reducing SUPs), perceived behavioral control (the degree to which individuals feel in control of reducing SUPs), and intention to reduce SUPs use in food packaging. The Likert scale used for this study ranges from 1 (strongly disagree) to 5 (strongly agree) to gauge participant agreement or disagreement with the subject matter. The instrument development was informed by recent publications on waste management behaviors [50, 51].

#### TTM measures (readiness to reduce SUPs consumption scale)

We developed the readiness to reduce SUP consumption scale using the University of Rhode Island Change Assessment (URICA) [52]. This scale consists of 32 items divided into four subscales: Pre-Contemplation, Contemplation, Action, and Maintenance. The items were translated from English into Thai by the research staff and then back-translated into English by an independent bilingual translator. Participants responded to all items on a 5-point Likert scale, ranging from strongly disagree (1) to strongly agree (5). The scale provides four separate stage scores and a composite readiness score, which was calculated by subtracting the pre-contemplation subscale mean scores from the sum of the contemplation, action, and maintenance subscale mean scores ([C + A + M]—PC) [53, 54].

We conducted reliability and validity tests on the questionnaire before converting it to an online format using Google Forms. To ensure content validity, a panel of experts in environmental science, environmental psychology, and public health reviewed a draft version of the questionnaire and provided feedback. Based on their comments, modifications were made before the questionnaire was used for data collection. A pilot study was also conducted with a small sample of participants (n = 35) to assess the questionnaire’s internal consistency. The results showed that the questionnaire subscales were sufficiently reliable, with Cronbach’s alpha coefficients ranging from 0.71 to 0.93 (Table 2). Four items of the modified URICA did not meet the threshold of the Cronbach alpha coefficient and were dropped from the main study.

**Table 2:** Instrument measurements.

Variables	Measurement
**Attitudes (ATT) (α = 0.91)**	
	I think reducing SUPs are necessary to protect the environment.
	I think SUPs are bad for the environment (especially the coastal areas).
	I think SUPs in food packaging are harmful to human health.
	I think reducing SUPs are worthwhile by using reusable alternatives.
**Subjective Norms (SN) (α = 0.82)**	
	My family and friends will be pleased to see me reuse SUPs.
	Most of my lecturers would approve of me paying to use reusable alternatives to SUPs.
	I use SUPs to gain the approval of my classmates.
**Perceived Behavioral Control (PBC) (α = 0.94)**	
	It is simple for me to turn down free SUPs in favor of reusable alternatives.
	I can always reuse alternatives to SUPs.
	Reducing the use of SUPs completely depends on me.
	It is easy for me to reduce SUPs usage.
**TTM**	
**Precontemplation (PC) (α = 0.73)**	
	As far as I am concerned, I do not have any problems regarding SUPs that need changing.
	It does not make sense to me to reduce SUP consumption.
	Being here is pretty much a waste of time for me because reducing SUPs doesn’t have to do with me.
	There is nothing that I need to change regarding SUP reduction.
	I may be part of the plastic pollution, but I do not think I am.
	All these talks about plastic pollution are boring; why can’t people just forget about this.
	I do not need to spend time thinking about SUP reduction.
**Contemplation (C) (α = 0.97)**	
	I think I might be ready for SUPs reduction.
	It might be worthwhile to work on my problems regarding SUPs use.
	I have been thinking that I might want to use reusable alternatives.
	I am hoping that environmental education will help me to understand SUPs reduction.
	I have a problem regarding SUPs and think I should work on it.
	I wish I had more ideas on how to reduce SUPs consumption
	I hope someone here will have some good advice for me.
**Action (A) (α = 0.87)**	
	I am doing something about reducing SUPs consumption that has been bothering me.
	I am finally doing some work to avoid excessive packaging with SUPs.
	At times SUPs reduction is difficult, but I am working on it.
	I am working hard to reduce SUPs in food packaging.
	I am working on using reusable alternatives to SUPs.
	I have started working on reducing SUPs, but I would like some help.
	Anyone can talk about SUPs reduction, but I am doing something about it.
	I am actively working on reducing SUPs.
**Maintenance (M) (α = 0.75)**	
	It worries me that I may go back to using SUPs I have already stopped, so I am here to seek help.
	I have been successful in using reusable alternatives to SUPs, but I am not sure I can keep up the effort on my own.
	I am not following through with what I had already changed regarding SUPs as I had hoped, and I am here to prevent a relapse.
	I thought once I start using alternatives to SUPs, I would be free of SUPs, but sometimes I still find myself struggling with them.
	I may need a boost right now to help me maintain the changes I have already made regarding SUPs.
	I am here to prevent myself from having a relapse of my problems.
**Behavioral Intention (BI) (α = 0.71)**	
	I intend to reduce SUP usage soon.
	I will pay extra to use reusable alternatives while shopping.
	I intend to avoid SUPs as much as I can while shopping or buying.
	I will give up SUPs for reusable alternatives.

The study sample consisted of both male (45.3%) and female students (54.7%) from the selected universities, with an average age of 20.31 years (SD = 3.21). Regarding their current academic year, 35.3% of the students were in their final year, while the remaining 64.7% were either in their program’s first, second, or third year. In terms of behavior change, 21.2% of study participants were in the pre-contemplation phase (phase 1), 52.1% in the contemplation phase (phase 2), 17.6% in the action phase (phase 3), and 9.1% in the maintenance phase (phase 4). In our study, the data collected from participants in the first phase corresponds to their responses on readiness to reduce SUP consumption, assessed using the TTM measures. The same individuals cannot indeed be in different phases of readiness to change, which is precisely the essence of the TTM.

### 2.3 Data analysis

To assess the adequacy of the model, we conducted Confirmatory Factor Analysis (CFA), examining various aspects such as goodness-of-fit, discriminant validity, convergent validity, and reliability. The model was evaluated by testing its goodness-of-fit using several indices, including the normed Chi-square (χ2) to degree of freedom (df) ratio, comparative-fit index (CFI), Normed Fit Index (NFI) and Root Mean Squared Error Approximation (RMSEA) [55]. Additionally, causal relationships among the variables within the model were examined. Given that the main concern was to test differences between groups, multigroup structural equation modeling (SEM) was used to analyze the influences of TPB on the intention to reduce SUP in different phases. As a result, respondents in one of the first three phases were considered [40]. All determinants (attitude, subjective norm, perceived behavioral control) were listed as predictors of behavioral intention. Data analysis was performed using IBM AMOS 23 software. Furthermore, we used Cohen’s rules of thumb to evaluate effect sizes to assess the result’s relevance beyond statistical significance. Our SEM analysis takes into account these different stages by considering the individual’s readiness score, which combines contemplation, action, and maintenance stages, and subtracts pre-contemplation scores, as per the URICA formula ([C + A + M]—PC). This composite readiness score was used as a latent variable in our SEM to explore how it relates to the TPB constructs and SUP reduction behaviors. In the SEM analysis, we examined how the composite readiness score, derived from the TTM measures, relates to the TPB constructs and SUP reduction behaviors. Specifically, we assessed how attitudes, subjective norms, perceived behavioral control, and intention (from TPB) influence the readiness score and subsequently impact SUP reduction behaviors.

### 2.4 Institutional review board statement

The study was conducted in accordance with the Declaration of Helsinki and approved by the Institutional Review Board (or Ethics Committee) of Chulalongkorn University (COA No. 032/66 and February 10, 2023). Informed consent was also obtained from all subjects involved in the study.

## 3.0 Results

### 3.1 Measurement model

The study’s findings indicated that the data was adequately fit by the CFA model, with a relative or normed Chi-square (χ^2^/df) value of 2.913 (p < 0.001). The RMSEA was 0.06, CFI was 0.991, and NFI was 0.940. Table 3 displays all significant factor loadings at or above 0.70. The study also found satisfactory internal consistency reliability and convergent validity levels, as evidenced by composite reliability (CR) and average variance extracted (AVE) values exceeding 0.7 and 0.5, respectively, as per references [56].

**Table 3:** The CFA results.

Constructs	Items	Loadings (λ)	CR	AVE	Mean ± SD
**ATT**			0.87	0.79	3.10 ± 1.13
	ATT1	0.851			
	ATT2	0.771			
	ATT3	0.887			
	ATT4	0.721			
**SN**			0.83	0.69	3.74 ± 1.32
	SN1	0.732			
	SN2	0.810			
	SN3	0.882			
**PBC**			0.91	0.76	2.26 ± 1.29
	PBC1	0.776			
	PBC2	0.901			
	PBC3	0.891			
	PBC4	0.905			
**PC**			0.88	0.80	
	PC1	0.754			2.94 ± 1.36
	PC2	0.876			
	PC3	0.881			
	PC4	0.711			
	PC5	0.889			
	PC6	0.743			
	PC7	0.749			
**C**			0.89	0.73	
	C1	0.873			3.90 ± 1.41
	C2	0.921			
	C3	0.857			
	C4	0.815			
	C5	0.887			
	C6	0.900			
	C7	0.811			
**A**			0.93	0.79	
	A1	0.817			2.38 ± 1.19
	A2	0.886			
	A3	0.834			
	A4	0.775			
	A5	0.875			
	A6	0.791			
	A7	0.780			
	A8	0.874			
**M**			0.85	0.70	
	M1	0.889			2.74 ± 1.51
	M2	0.879			
	M3	0.849			
	M4	0.895			
	M5	0.880			
	M6	0.890			
**BI**			0.90	0.78	
	BI1	0.779			3.97 ± 1.21
	BI2	0.830			
	BI3	0.911			
	BI4	0.951			

### 3.2 Analysis of students’ motivations and phase of change regarding SUP reduction

The study used the TPB and TTM to investigate single-use plastic consumption reduction among the selected students, as presented in Table 3. The findings suggest that the students held positive attitudes towards reducing their single-use plastic consumption, with a mean of 3.10 and a standard deviation (SD) of 1.13. Subjective norms, which reflect perceived social pressure to engage in the behavior, had a relatively high mean score of 3.74 (SD = 1.32), indicating the importance of social influence on students’ behavior. In contrast, perceived behavioral control, which refers to the perceived ease or difficulty of performing the behavior, had a relatively low mean score of 2.26 (SD = 1.29), suggesting potential obstacles to changing behavior.

Regarding TTM constructs, most students were in the contemplation phase (Mean = 3.90, SD = 1.41), meaning they were considering reducing their single-use plastic consumption but had not taken action yet. However, fewer students had started to change their behavior, as indicated by a relatively low mean score for the action phase of 2.38 (SD = 1.19). The maintenance phase had a mean score of 2.74 (SD = 1.51), implying that students who had changed their behavior may struggle to maintain the changes in the long term. Finally, the study found that the mean score for the pre-contemplation phase of TTM was 2.94 (SD = 1.36).

### 3.3 Structural equation modelling: Hypothesis testing results

The CFA results demonstrated a good fit to the data, with a normed Chi-square (χ^2^/df) value of 2.97 (p < 0.001), indicating that the social-psychological factors explained more than 30% of the variance in all three phases of the study. The model fit indices were acceptable, with a CFI of 0.970, RMSEA of 0.067, and NFI of 0.928 [57]. As per Cohen’s guidelines, the effect sizes of the relationships between the psychosocial factors and behavioral intention were generally small to medium [58].

**Fig 2:**
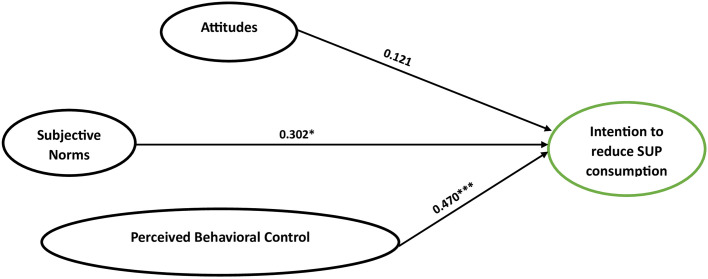
The multi-group analysis of phase 1 variables.

The multi-group analysis for students in phase 1 revealed significant positive relationships between perceived behavioral control and subjective norm with behavioral intention (Table 4). The analysis revealed that attitude did not significantly affect phase 1. In line with Cohen’s guidelines [58], which classify effect sizes as small (0.1), medium (0.3), and large (0.5), our findings suggested that subjective norms and perceived behavioral control exhibited effect sizes that are closer to medium (Fig 2).

**Table 4:** Multigroup structural equation models based on the phase of behavior change (n = 317).

Constructs	Phase 1		Phase 2		Phase 3	
	β	S.E.	β	S.E.	β	S.E.
**ATT → BI**	0.121	0.057	0.138*	0.073	0.261	0.110
**SN → BI**	0.302*	0.033	0.174	0.105	0.187	0.028
**PBC → BI**	0.470***	0.150	0.683***	0.082	0.315***	0.152

Notes:

***p < 0.001;

*p < 0.05, β = Standardized Estimates, S.E. = Standard Error

**Fig 3:**
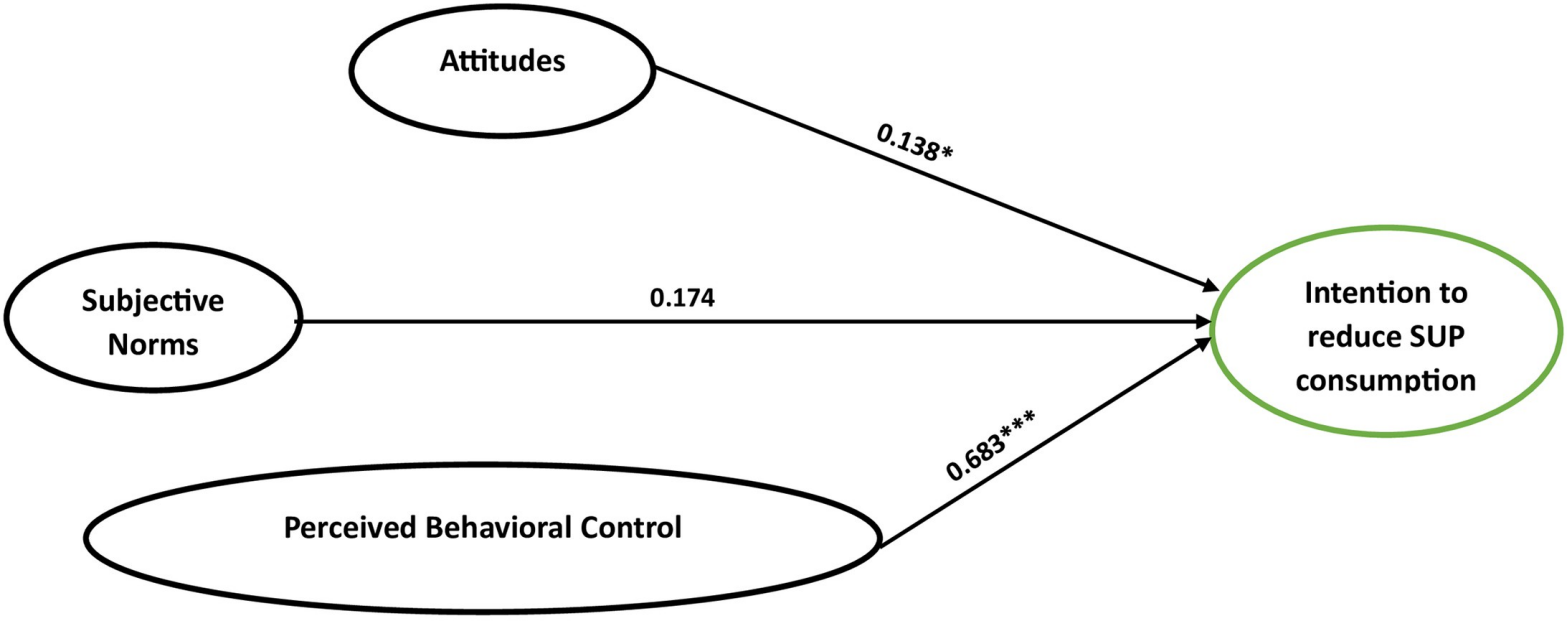
The multi-group analysis of phase 2 variables.

For students in phase 2, significant positive relationships existed between attitude, perceived behavioral control and behavioral intention (Table 4). The effect sizes for attitude and subjective norm were small, while perceived behavioral control was medium (Fig 3).

**Fig 4:**
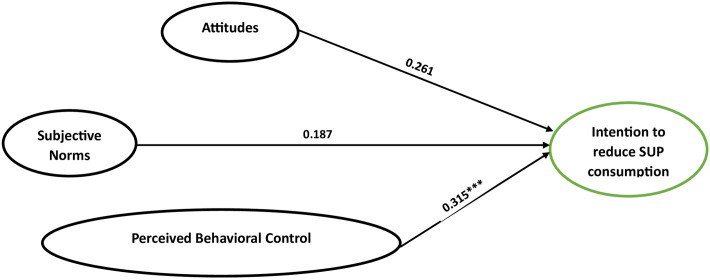
The multi-group analysis of phase 3 variables.

The multi-group SEM analysis showed a positive influence of perceived behavioral control on behavioral intention, confirming H3 (Table 4). Per Cohen’s guidelines, the effect size of perceived behavioral control on behavioral intention was large [58] as shown in Fig 4.

To test H4, we conducted a pairwise path coefficient difference test for perceived behavioral control across all phases. Specifically, we compared the coefficients for the effects of perceived behavioral control on the behavioral intention for phases 1, 2, and 3. The critical ratios for a t-test are provided in Table 5 (where a value greater than an absolute value of 1.96 indicates a statistically significant difference at p < 0.05). The results indicated significant differences in the relationship between perceived behavioral control and behavioral intention across the phases (**PBC**_**Phase3**_ **→ BI and PBC**_**Phase2**_ **→ BI),** thus supporting H4.

**Table 5:** Pairwise parameter comparison for H4.

	**PBC**_**Phase1**_ **→ BI**	**PBC**_**Phase2**_ **→ BI**
**PBC**_**Phase2**_ **→ BI**	0.914	
**PBC**_**Phase3**_ **→ BI**	2.137	1.985

## 4.0 Discussion

The study provides new insights into the dynamics of decision-making for pro-environmental behavior by examining the influence of psychosocial factors on behavioral intentions towards SUP reduction among Thai university students. Specifically, the study adopts a dynamic approach to the Theory of Planned Behavior (TPB) and explores how attitudes, perceived behavioral control, and subjective norms affect SUP reduction intention at different phases of the behavior change process. The research contributes to the existing literature on pro-environmental behavior in two ways: by shedding light on the dynamic process of behavior change and adding to the knowledge regarding pro-environmental behavior in the context of plastic waste management.

The current study investigates the impact of psychosocial factors on the intention to reduce SUP consumption, in line with the stages of behavior change. Our results revealed that attitudes only predicted behavioral intentions among individuals in the contemplation phase of reducing SUP, meaning they have considered reducing SUP consumption but have not taken action yet. This finding confirms earlier research on sustainable mobility and energy-efficient homes and extends its applicability to plastic waste management [40]. It also provides insights into the study findings on the students’ SUP reduction behavior, indicating that attitudes influence behavioral intentions only when individuals are in the contemplation phase of the behavior change process [59]. Specifically, attitudes are a significant predictor of behavioral intentions only when the students have considered reducing plastic consumption but have not yet done so. This implies that attitudes do not influence the behavioral intentions of the students who have already taken action [39, 40]. However, the effect size for attitudes is small, suggesting a limited influence on behavioral intentions to reduce SUP among the students. This is consistent with criticisms of the prior TPB-based research reporting only a small influence of attitudes on pro-environmental behavior [60, 61]. This implies that attitudes alone may not be the primary driver for individuals who have already taken action to reduce SUP consumption. This phenomenon can be attributed to the idea that individuals who have reached the action phase may be motivated by factors beyond attitudes, such as habituation, perceived effectiveness of their actions, or external incentives. This highlights the importance of considering different motivational factors at various stages of behavior change.

Furthermore, we found that perceived behavioral control significantly impacts behavioral intentions across all three phases of behavior change, highlighting its importance in the context of SUP reduction. This result is consistent with previous research on pro-environmental behavior [39, 40]. However, it is a novel insight in the context of waste management and adds to existing knowledge by emphasizing the significance of people’s perceptions of their abilities and opportunities to reduce plastic use. To our knowledge, no study has investigated the influence of perceived behavioral control in this context. Our results indicated that the effect of perceived behavioral control is equally strong for individuals in all phases of behavior change based on significance testing, but effect sizes suggest a more significant effect in the second phase of behavior change. The consistent and strong influence of perceived behavioral control across all three phases of behavior change emphasizes its significance in the context of SUP reduction. This suggests that individuals’ perceptions of their abilities and opportunities to reduce plastic use play a crucial role in shaping their intentions. The stronger effect size observed in the second phase of behavior change may be linked to the transition from contemplation to action, where individuals actively assess their control over their behavior and its feasibility.

The present study supports our theoretical predictions that subjective norms positively influence the students’ behavioral intentions in the pre-contemplation phase of SUP reduction behavior. Recent research in environmental psychology and housing has also emphasized the importance of normative factors in promoting pro-environmental behavior [44, 62]. However, the effect sizes of these factors on SUP reduction intention are relatively small. While subjective norms positively influence behavioral intentions in the pre-contemplation phase, the relatively small effect sizes indicate that normative factors may have a limited impact on SUP reduction intention. Possible causes for this phenomenon could include the presence of competing social influences or the need for more targeted normative interventions. This finding is consistent with previous research highlighting the limited influence of normative factors on pro-environmental behavior [44, 60].

Furthermore, our findings regarding the phase-dependent influence of these factors can contribute to a more comprehensive understanding of the contradictory findings in empirical studies that rely on the Theory of Planned Behavior. As previous studies have pointed out, incorporating phase models of behavior change into the Theory of Planned Behavior may help explain the inconsistencies observed in earlier research [39]. The inconsistencies deal with variations in the predictive power of TPB constructs across different studies [32, 35, 63], particularly regarding pro-environmental behavior. Therefore, incorporating phase models, such as the Transtheoretical Model (TTM), allows for a nuanced understanding of when and how psychosocial factors impact behavioral intentions. By examining attitudes, perceived behavioral control, and subjective norms at different phases of behavior change, our study provides insights into the contextual variability of these factors and their influence on SUP reduction intentions. This approach helps explain why attitudes, for instance, may have a limited effect on individuals who have already taken action (consistent with prior TPB-based research) while remaining influential for those still contemplating behavior change.

In our exploration of pro-environmental behavior, particularly in the realm of SUP reduction, an essential facet that warrants consideration is the concept of “behavioral spillover”. Behavioral spillover refers to the phenomenon where the adoption of one sustainable behavior influences the adoption of additional environmentally friendly practices. While our study primarily scrutinizes the stages of behavior change in the context of SUP reduction, it is pertinent to examine the potential spillover effects on behavioral maintenance and other pro-environmental actions. Our findings emphasize the significance of understanding how attitudes, perceived behavioral control, and subjective norms operate differently across various phases of SUP reduction readiness. This insight raises the question of whether fostering positive attitudes and enhancing perceived behavioral control in the pursuit of SUP reduction may trigger broader behavioral spillover effects.

Behavioral maintenance, beyond the initial adoption of SUP reduction practices, is crucial for sustained environmental impact. Integrating behavioral spillover into our discussion allows us to explore how successful SUP reduction initiatives might catalyze a ripple effect, leading individuals to embrace and maintain other eco-friendly behaviors. For instance, the positive attitudes developed during the contemplation phase of SUP reduction may extend to increased recycling efforts or reduced overall plastic consumption. In considering the literature on behavioral spillover, exemplified by studies like Ibanez and Roussel [64] and Lanzini and Thøgersen [65], we recognize the interconnected nature of pro-environmental behaviors. Addressing behavioral spillover in the context of SUP reduction offers a pathway to comprehensively understand the long-term impact of interventions.

### 4.1 Theoretical implications

Our study significantly advances theoretical understanding by showcasing the adaptability of the TTM within the intricate domain of plastic waste management. Specifically, our research delves into the multifaceted relationship between TTM and the stages of behavior change in the context of SUP reduction. In doing so, we discern that key determinants—attitudes, perceived behavioral control, and subjective norms—exhibit dynamic variations across distinct phases of SUP reduction readiness. The revelation of these nuanced dynamics offers a distinctive theoretical lens through which the interplay of psychosocial factors and behavior change can be comprehended. This insight propels the theoretical foundation beyond the confines of traditional frameworks such as the TPB and TTM. We not only affirm the relevance of these theories but also elucidate their integrative potential, fostering a more holistic understanding of pro-environmental behavior.

Our findings underscore that attitudes predominantly influence behavioral intentions during the contemplation phase of SUP reduction, revealing a phase-specific impact. This nuanced perspective challenges prior assumptions and aligns with recent calls for a more contextualized examination of psychosocial factors. Moreover, the consistently significant role of perceived behavioral control across all phases highlights its universal importance in shaping intentions for SUP reduction. By highlighting the dynamic interplay of psychosocial factors across behavior change phases, our study opens avenues for the integration of diverse behavior change theories. This not only expands the theoretical landscape but also provides a conceptual framework to guide future research in pro-environmental behavior. The intricate dance between TTM, TPB, and the contextual dynamics of SUP reduction readiness serves as a stepping stone for researchers and practitioners seeking a more nuanced and encompassing perspective on sustainable behavior change. Our theoretical contributions extend beyond the immediate scope of this study, fostering a paradigm shift towards an integrative and contextually sensitive understanding of pro-environmental behavior.

### 4.2 Practical implications

Based on our empirical research, the fundamental recommendation for practitioners is to segment residents according to the phase of behavior change they have reached. Overall, we must assume that campaigns that address subjective norms are only effective for those who have not yet thought about reducing SUP consumption (phase 1). Given that the largest proportions of the study population, at least from the Thai universities we investigated, fall into phase 2 **(51.2%),** these strategies seem most recommended in practice. For instance, campus initiatives can engage students and youth leaders to actively participate in sharing their personal SUP reduction experiences and sustainable lifestyle tips through popular social media platforms such as Line, Twitter, TikTok, or Instagram. These platforms can effectively spread awareness and foster a culture of SUP reduction among students. Offline workshops and events can also be organized to complement online efforts, providing opportunities for disseminating normative information and encouraging peer-to-peer interactions. Considering effect sizes, a promising strategy seems to be to influence perceived behavior control, particularly for students in phases 1 and 2. Those individuals are probably best reached through service offers such as informational campaigns providing procedural knowledge on how to adopt reusable alternatives to SUP. For instance, educational programs can leverage virtual reality (VR) technology to create simulated scenarios regarding SUP reduction initiatives. Through immersive experiences, students can actively engage in these activities and develop practical skills by “doing” in a virtual environment. This interactive approach provides a hands-on learning experience that enhances their understanding of SUP reduction practices. By utilizing VR technology, reduction initiatives can offer a unique and engaging way to educate and empower students in their SUP reduction efforts.

If decision-makers wish to address an audience that has considered reducing SUP consumption but has not yet done so (individuals belonging to phase 2), they might also consider using informational campaigns to influence public attitudes. This includes communication campaigns that convey factual information about, for example, the positive/negative effects of reducing/using SUP on the environment. With phase-targeted campaigns, public policymakers can allocate marketing/training funds more efficiently and presumably with greater effectiveness.

### 4.3 Study limitations and direction for future studies

It should be noted that the findings of this study, which focused on university students from Thailand, may not be readily generalizable to the broader population, such as residents or individuals in individualistic cultures. The sample of university students represents a subset of young adults with similar age and environmental learning experiences. Consequently, they may possess a heightened understanding of the environmental system and hold stronger environmental attitudes than the general population. This discrepancy may result in varying patterns concerning the relationships between attitudes, subjective norms, perceived behavioral control and intention to reduce SUP between these two population groups. Future studies should consider the general population in their analysis.

Moreover, the study participants were recruited using an online, non-probabilistic sampling method, which may have resulted in self-selection bias. This could limit the generalizability of the study’s findings. To address this limitation, future studies could consider using more diverse samples and probabilistic sampling methods to improve the generalizability of the results. Additionally, using self-reported measures in this study may introduce response bias. Future research could incorporate objective measures or multiple data collection methods to validate self-reported measures. Another area for future research could be to expand the scope of the study beyond SUP reduction behavior and include other pro-environmental behaviors. This study highlights the importance of various social-psychological factors in the behaviour change process when making decisions to reduce SUP consumption. However, additional research is necessary to confirm how these determinants vary in other waste management contexts, such as reuse, recycling, and conservation.

By acknowledging and exploring behavioral spillover within the context of SUP reduction, we suggest a holistic approach that considers the interconnected web of pro-environmental behaviors, fostering a more resilient and enduring commitment to sustainable living. So, future research and sustainability initiatives should delve into how encouraging SUP reduction can serve as a catalyst for sustained environmentally conscious actions, contributing not only to the reduction of single-use plastics but also to a broader shift toward sustainable living practices.

## 5.0 Conclusion

Given the public concerns about the adverse impact of plastic waste on the ecosystem, promoting SUP reduction among the youth is crucial. Therefore, it is essential to understand the psychological factors that influence students’ decision-making processes. Our findings illuminate the complexity of the behavior change process within the context of SUP reduction among university students. We identify four distinct phases; three of them is influenced by a unique set of psychosocial factors. This segmentation underscores the need for tailored interventions and strategies that align with the readiness levels of students. This insight enables us to devise more effective and targeted approaches for behavior change campaigns. Our study emphasizes the potential effectiveness of communication campaigns that highlight positive subjective norms, particularly for individuals in the initial phase of behavior change. By leveraging the influence of these norms, interventions can tap into the social dynamics that shape students’ decisions regarding SUP reduction. This insight offers an innovative approach to fostering pro-environmental behavior among this demographic. Perceived behavioral control emerges as a significant factor impacting intentions to reduce SUP consumption, particularly in the third phase of behavior change. This finding underscores the importance of investing in infrastructure and accompanying services to support individuals in this stage. Establishing a robust waste management system that aligns with students’ perceived control over their actions can be a crucial step toward achieving sustainable SUP reduction.

## Supporting information

S1 File (PDF)

S2 File (PDF)

S3 File (XLSX)
