# Supplementary material for: Breaking the plastic habit: Drivers of single-use plastic reduction among Thai university students
Source: PLoS One. 2024 May 9;19(5):e0299877. doi: 10.1371/journal.pone.0299877 (PMC11081253; doi:10.1371/journal.pone.0299877)
Supplement: S1 File — (PDF) [file pone.0299877.s001.pdf]

Breaking the plastic habit: Drivers of single-use plastic reduction among Thai university students

This study aims to identify attitudes, beliefs and motivational processes that might be useful in designing interventions to reduce SUPs food packaging among university students in Thailand. The participants will answer the questions based on an extended Theory of Planned Behavior (TPB) and the Transtheoretical Model (TTM) of change. All items will be measured on a 5-point Likert scale ranging from strongly disagree (1) to strongly agree (5).

Note: Single-use plastics (SUPs) food packaging are plastic materials meant to be used only once before they are thrown away. For this study, SUPs include grocery bags, food containers (such as takeaway plates), drinking containers (plastic bottles, disposable cups), straws, coffee stirrers, disposable cutlery (e.g., spoons, forks, knives) and plates, and produce bags. Also, for simplicity, SUPs' food packaging will be referred to as SUPs in this questionnaire.

This questionnaire is part of a research project focused on Thai people's use of single-use plastics. All information will be kept in strict confidentiality and will be used for research purposes only. Please continue if you agree to take part in this research.

A. Participants' details

1. Age _______?

2. Gender? ❑[1] Female ❑[2] Male

3. Department in the university ____________?

4. What is your current year of study___________?

S1 Table: TPB and TTM constructs

Variables	Items
**TPB**	
**Attitudes**	
	I think reducing SUPs are necessary to protect the environment.
	I think SUPs are bad for the environment (especially the coastal areas).
	I think SUPs in food packaging are harmful to human health.
	I think reducing SUPs are worthwhile by using reusable alternatives.
**Subjective Norms**	
	My family and friends will be pleased to see me reuse SUPs.
	Most of my lecturers would approve of me paying to use reusable alternatives to SUPs.
	I use SUPs to gain the approval of my classmates.
**Perceived Behavioral Control**	
	It is simple for me to turn down free SUPs in favor of reusable alternatives.
	I can always reuse alternatives to SUPs.
	Reducing the use of SUPs completely depends on me.
	It is easy for me to reduce SUPs usage.

**TTM**	
**Precontemplation**	
	As far as I am concerned, I do not have any problems regarding SUPs that need changing.
	It does not make sense to me to reduce SUP consumption.
	Being here is pretty much a waste of time for me because reducing SUPs doesn't have to do with me.
	There is nothing that I need to change regarding SUP reduction.
	I may be part of the plastic pollution, but I do not think I am.
	All these talks about plastic pollution are boring; why can't people just forget about this.
	I do not need to spend time thinking about SUP reduction.
**Contemplation**	
	I think I might be ready for SUPs reduction.
	It might be worthwhile to work on my problems regarding SUPs use.
	I have been thinking that I might want to use reusable alternatives.
	I am hoping that environmental education will help me to understand SUPs reduction.
	I have a problem regarding SUPs and think I should work on it.
	I wish I had more ideas on how to reduce SUPs consumption.
	I hope someone here will have some good advice for me.

**Action**	
	I am doing something about reducing SUPs consumption that has been bothering me.
	I am finally doing some work to avoid excessive packaging with SUPs.
	At times SUPs reduction is difficult, but I am working on it.
	I am working hard to reduce SUPs in food packaging.
	I am working on using reusable alternatives to SUPs.
	I have started working on reducing SUPs, but I would like some help.
	Anyone can talk about SUPs reduction, but I am doing something about it.
	I am actively working on reducing SUPs.
**Maintenance**	
	It worries me that I may go back to using SUPs I have already stopped, so I am here to seek help.
	I have been successful in using reusable alternatives to SUPs, but I am not sure I can keep up the effort on my own.
	I am not following through with what I had already changed regarding SUPs as I had hoped, and I am here to prevent a relapse.
	I thought once I start using alternatives to SUPs, I would be free of SUPs, but sometimes I still find myself struggling with them.

	I may need a boost right now to help me maintain the changes I have already made regarding SUPs.
	I am here to prevent myself from having a relapse of my problems.
**Behavioral Intention**	
	I intend to reduce SUP usage soon.
	I will pay extra to use reusable alternatives while shopping.
	I intend to avoid SUPs as much as I can while shopping or buying.
	I will give up SUPs for reusable alternatives.
