# Supplementary material for: Breaking the plastic habit: Drivers of single-use plastic reduction among Thai university students
Source: PLoS One. 2024 May 9;19(5):e0299877. doi: 10.1371/journal.pone.0299877 (PMC11081253; doi:10.1371/journal.pone.0299877)
Supplement: S2 File — (PDF) [file pone.0299877.s002.pdf]

ชื่อโครงการ: บรรจุภัณฑ์อาหารพลาสติกแบบใช้ครั้งเดียวทิ้งในประเทศไทย: การรับรู้ของสาธารณชน ความตั้งใจเชิงพฤติกรรม และแรงผลักดัน

คำถามเกี่ยวกับพฤติกรรมการลดบรรจุภัณฑ์อาหารพลาสติกแบบใช้ครั้งเดียวทิ้ง (SUP) ของชาวไทย

คำถามในการศึกษาวิจัยนี้คือ “แบบจำลองทฤษฎีพฤติกรรมตามแผน (TPB) รวมถึงปัจจัยสถานการณ์ และแบบจำลองทางทฤษฎี ค่านิยม ความเชื่อและบรรทัดฐาน (VBN) ที่อธิบายพฤติกรรมการลดบรรจุภัณฑ์อาหารพลาสติกแบบใช้แล้วทิ้งของคนไทย”

คำชี้แจงในการตอบแบบสอบถาม

แบบสอบถามมีทั้งหมด 4 ตอน ดังนี้

ตอนที่ 1 ข้อมูลทั่วไปของผู้ตอบแบบสอบถาม

ตอนที่ 2 คำถามประเมินความตั้งใจในการลดบรรจุภัณฑ์อาหารชนิดพลาสติกแบบใช้ครั้งเดียวทิ้งของนิสิต/นักศึกษามหาวิทยาลัยไทย

ตอนที่ 3 ความคิดเห็นและข้อเสนอแนะเพิ่มเติม

ตอนที่ 1 ข้อมูลทั่วไปของผู้ตอบแบบสอบถาม

<u>คำชี้แจง</u> โปรดเติมข้อความหรือตัวเลขและใส่ √ ลงในช่อง ☐ หน้าข้อความที่ตรงกับความเป็นจริงเกี่ยวกับตัวท่าน

1. อายุ.................ปี
2. เพศ ☐ ชาย ☐ หญิง ☐ อื่นๆ
3. คณะ...

   ภาควิชา...
4. ชั้นปีที่กำลังศึกษา ☐ 1 ☐ 2 ☐ 3 ☐ 4 ☐ มากกว่า 4

ตอนที่ 2 คำถามประเมินความตั้งใจในการลดบรรจุภัณฑ์อาหารชนิดพลาสติกแบบใช้ครั้งเดียวทิ้งของนิสิต/นักศึกษามหาวิทยาลัยไทย

<u>คำชี้แจง</u> โปรดใส่เครื่องหมาย √ ลงในช่องที่ตรงกับความคิดเห็นของท่านมากที่สุด โดยกำหนดคำตอบเป็นคะแนน 5 ระดับ คือ

5 หมายถึง เห็นด้วยอย่างยิ่ง

4 หมายถึง เห็นด้วย

3 หมายถึง เป็นกลาง

2 หมายถึง ไม่เห็นด้วย

1 หมายถึง ไม่เห็นด้วยอย่างยิ่ง

ประเด็นคำถาม	ระดับความคิดเห็น				
	1	2	3	4	5
**ส่วนที่ 1 ทฤษฎีพฤติกรรมตามแผน (Theory of Planned Behavior : TPB)**					
**ทัศนคติ (Attitudes)**					
1. ฉันคิดว่าการลดพลาสติกแบบใช้ครั้งเดียวทิ้งมีความสำคัญในการช่วยรักษาสิ่งแวดล้อม					
2. ฉันคิดว่าพลาสติกแบบใช้ครั้งเดียวทิ้งนั้นส่งผลเสียต่อสิ่งแวดล้อม (โดยเฉพาะสิ่งแวดล้อมบริเวณชายฝั่ง)					
3. ฉันคิดว่าบรรจุภัณฑ์อาหารพลาสติกแบบใช้ครั้งเดียวทิ้งเป็นอันตรายต่อสุขภาพของมนุษย์					
4. ฉันคิดว่าการลดพลาสติกแบบใช้ครั้งเดียวทิ้งด้วยการใช้บรรจุภัณฑ์ทางเลือกที่ใช้ซ้ำได้นั้นคุ้มค่า					
**การคล้อยตามกลุ่มอ้างอิง (Subjective Norms)**					
1. ครอบครัวและเพื่อน ๆ ของฉันยินดีที่เห็นฉันนำบรรจุภัณฑ์อาหารประเภทพลาสติกแบบใช้ครั้งเดียวทิ้งกลับมาใช้ซ้ำใหม่					
2. อาจารย์ของฉันส่วนใหญ่เห็นด้วยกับการที่ฉันซื้อบรรจุภัณฑ์ทางเลือกที่ใช้ซ้ำได้แทนพลาสติกแบบใช้ครั้งเดียวทิ้ง					
3. ฉันใช้บรรจุภัณฑ์อาหารประเภทพลาสติกใช้ครั้งเดียวทิ้ง เพื่อให้ได้รับการยอมรับจากเพื่อน ๆ					
**การรับรู้ความสามารถในการควบคุมพฤติกรรม (Perceived Behavioral Control)**					
1. เป็นเรื่องง่ายมากสำหรับฉันที่จะปฏิเสธพลาสติกแบบใช้ครั้งเดียวทิ้ง (แจกฟรี) แล้วหันมาใช้บรรจุภัณฑ์ทางเลือกที่นำกลับมาใช้ซ้ำได้					
2. ฉันสามารถใช้บรรจุภัณฑ์ทางเลือกอื่นแทนการใช้บรรจุภัณฑ์อาหารประเภทพลาสติกใช้ครั้งเดียวทิ้งได้เสมอ					
3. การลดการใช้พลาสติกแบบใช้ครั้งเดียวทิ้งได้อย่างสมบูรณ์นั้นขึ้นอยู่กับตัวฉัน					
4. มันง่ายสำหรับฉันที่จะลดการใช้บรรจุภัณฑ์อาหารประเภทพลาสติกใช้ครั้งเดียวทิ้ง					

ประเด็นคำถาม	ระดับความคิดเห็น				
	1	2	3	4	5
**ส่วนที่ 2 การปรับเปลี่ยนพฤติกรรม (Transtheoretical Model: TTM)**					
**การเมินเฉย ไม่คิดว่ามีปัญหา (Precontemplation)**					
1. เท่าที่ฉันทราบ ฉันไม่มีปัญหาอะไรกับการใช้พลาสติกแบบใช้ครั้งเดียวทิ้ง ซึ่งมีความจำเป็นต้องเปลี่ยนแปลง					
2. มันไม่สมเหตุสมผลสำหรับฉันที่ต้องลดการบริโภคพลาสติกแบบใช้ครั้งเดียวทิ้ง					
3. สำหรับฉัน การลดพลาสติกแบบใช้ครั้งเดียวทิ้งมันค่อนข้างเป็นเรื่องเสียเวลาเพราะไม่เกี่ยวข้องอะไรกับฉัน					
4. ไม่มีอะไรเกี่ยวกับพลาสติกแบบใช้ครั้งเดียวทิ้งที่ฉันจำเป็นต้องเปลี่ยนแปลง					
5. ฉันอาจเป็นส่วนหนึ่งของปัญหามลพิษพลาสติก แต่ฉันไม่คิดว่าฉันมีส่วนเกี่ยวข้อง					
6. การบรรยายเกี่ยวกับมลพิษพลาสติกทั้งหมดน่าเบื่อ ทำไมผู้คนไม่ลืม ๆ มันไปเลย					
7. ฉันไม่จำเป็นต้องใช้เวลาในการคิดถึงเรื่องการลดพลาสติกแบบใช้ครั้งเดียวทิ้ง					
**ตระหนักรู้ว่ามีปัญหา/การไตร่ตรอง (Contemplation)**					
1. ฉันคิดว่าฉันจะลดการใช้พลาสติกแบบใช้ครั้งเดียวทิ้ง					
2. เป็นสิ่งที่คุ้มค่าที่ฉันจะลดการใช้พลาสติกแบบใช้ครั้งเดียวทิ้ง					
3. ฉันคิดว่าฉันควรใช้บรรจุภัณฑ์ทางเลือกที่สามารถนำกลับมาใช้ซ้ำได้					
4. ฉันหวังว่าการศึกษาเกี่ยวกับสิ่งแวดล้อมจะช่วยให้ฉันมีความเข้าใจเกี่ยวกับการลดพลาสติกแบบใช้ครั้งเดียวทิ้ง					
5. ฉันสร้างปัญหาจากการใช้พลาสติกแบบใช้ครั้งเดียวทิ้ง และคิดว่าฉันเองก็ควรแก้ไขปัญหานี้					
6. ฉันหวังว่าฉันจะมีแนวคิดเพิ่มเติมเกี่ยวกับวิธีลดการใช้พลาสติกแบบใช้ครั้งเดียวทิ้ง					
7. ฉันหวังว่าจะมีใครสักคนที่จะให้คำแนะนำที่ดีสำหรับฉันได้					

ประเด็นคำถาม	ระดับความคิดเห็น				
	1	2	3	4	5
**การปรับเปลี่ยน (Action)**					
1. ฉันกำลังลดการใช้พลาสติกแบบใช้ครั้งเดียวทิ้ง					
2. ฉันกำลังหลีกเลี่ยงการใช้บรรจุภัณฑ์พลาสติกแบบใช้ครั้งเดียวทิ้งที่มากเกินไป					
3. บางครั้งการลดการใช้พลาสติกแบบใช้ครั้งเดียวทิ้งเป็นเรื่องที่ยาก แต่ฉันเองก็กำลังทำอยู่					
4. ฉันตั้งใจเต็มที่เพื่อลดการใช้บรรจุภัณฑ์อาหารประเภทพลาสติกแบบใช้ครั้งเดียวแล้วทิ้ง					
5. ฉันกำลังพยายามใช้พลาสติกทางเลือกอื่น ๆ ที่สามารถนำกลับมาใช้ซ้ำได้แทนการใช้พลาสติกแบบใช้ครั้งเดียวทิ้ง					
6. ฉันกำลังเริ่มลดการใช้พลาสติกแบบใช้ครั้งเดียวทิ้ง...แต่ฉันก็ยังต้องการความช่วยเหลืออยู่					
7. ในขณะที่ทุกคนพูดถึงการลดการใช้พลาสติกแบบใช้ครั้งเดียวทิ้ง ฉันเองก็กำลังเปลี่ยนแปลงตัวเองเพื่อลดการใช้พลาสติกแบบใช้ครั้งเดียวทิ้ง					
8. ฉันกำลังลดการใช้บรรจุภัณฑ์อาหารประเภทพลาสติกแบบใช้ครั้งเดียวทิ้งอย่างจริงจังและตั้งใจ					
**การคงไว้ซึ่งการปรับเปลี่ยน (Maintenance)**					
1. ฉันกังวลว่าฉันอาจจะกลับไปใช้พลาสติกแบบใช้ครั้งเดียวทิ้งอีกครั้ง ทั้งที่ฉันได้หยุดใช้ไปแล้ว ซึ่งฉันต้องการความช่วยเหลือ					
2. ฉันประสบความสำเร็จในการใช้บรรจุภัณฑ์ทางเลือกแบบใช้ซ้ำแทนการใช้พลาสติกแบบใช้ครั้งเดียวทิ้ง แต่ฉันยังไม่แน่ใจว่าจะสามารถพยายามทำต่อไปได้อีกหรือเปล่า					
3. ฉันไม่สามารถลดการใช้พลาสติกแบบใช้ครั้งเดียวทิ้งอย่างที่ตั้งใจไว้ได้...และฉันหวังว่าฉันจะไม่ทำให้เกิดเหตุการณ์เช่นนี้อีก					
4. ฉันคิดว่าเมื่อฉันเริ่มใช้บรรจุภัณฑ์ทางเลือกอื่นๆแทนการใช้พลาสติกแบบใช้ครั้งเดียวทิ้ง ฉันก็จะไม่มีการใช้พลาสติกแบบใช้ครั้งเดียวอีก แต่ในบางครั้งก็ยังพบว่าตัวเองต้องการใช้พลาสติกประเภทนี้อยู่บ้าง					
5. ฉันต้องการแรงกระตุ้นเพื่อช่วยให้ฉันสามารถลดการใช้บรรจุภัณฑ์อาหารประเภทพลาสติกแบบใช้ครั้งเดียวทิ้งได้ต่อไป					
6. ฉันยืนยันที่จะไม่ใช้พลาสติกแบบใช้ครั้งเดียวทิ้งอีก					

ประเด็นคำถาม	ระดับความคิดเห็น				
	1	2	3	4	5
**ความตั้งใจในการเปลี่ยนแปลงพฤติกรรม (Behavioral Intention)**					
1. ฉันตั้งใจจะลดการใช้บรรจุภัณฑ์อาหารประเภทพลาสติกแบบใช้ครั้งเดียวทิ้ง ในไม่ช้านี้					
2. ฉันจ่ายเงินเพิ่มเพื่อซื้อบรรจุภัณฑ์ทางเลือกที่นำกลับมาใช้ซ้ำได้ในการช้อปปิ้ง/ซื้อสินค้า					
3. ฉันตั้งใจที่จะหลีกเลี่ยงการใช้พลาสติกแบบใช้ครั้งเดียวทิ้งเท่าที่ฉันทำได้ในการช้อปปิ้ง/ซื้อสินค้า					
4. ฉันจะเลิกใช้พลาสติกแบบใช้ครั้งเดียวทิ้ง โดยหันมาใช้บรรจุภัณฑ์อาหารทางเลือกที่นำกลับมาใช้ซ้ำได้					

ตอนที่ 3 ความคิดเห็นและข้อเสนอแนะเพิ่มเติม

....

....

....

....

ขอขอบคุณทุกท่านที่ได้มีส่วนร่วมในการให้ข้อมูล
